# Parallel study about the effects of psychotherapy on patients with dental phobia determined by anxiety scores and saliva secretion and composition

**DOI:** 10.1186/s12903-016-0264-2

**Published:** 2016-08-02

**Authors:** E. A. Naumova, S. Faber, P. Lindner, A. Wannemueller., T. Sandulescu, P. Joehren, W. H. Arnold

**Affiliations:** 1 Department of Biological and Material Sciences in Dentistry, School of Dentistry, Faculty of Health, Witten/Herdecke University, Witten, Germany; 2 Dental Clinic Bochum, Bochum, Germany

**Keywords:** Dental phobia, Salivary cortisol, Salivary protein, Dental anxiety score, State trait anxiety score, Psychotherapy

## Abstract

**Background:**

The aim of this study was to determine the success of psychotherapeutic treatment for dental phobia by measurement of anxiety using the dental anxiety score (DAS), the state trait anxiety score (STAI state), salivary cortisol and protein concentrations and the salivary secretion rate. Primary endpoint of the study was the comparison of the data before and after psychotherapeutic treatment.

**Methods:**

Forty patients were included into the study. Twenty-four were allocated to the phobic group, 16 to the control group. Saliva was collected upon entering the dental clinic and again after three weeks of psychotherapy. The results were compared with those of a control group. The DAS and STAI questionnaires were completed at each visit.

**Results:**

A reduction in DAS values was found after psychotherapy. However, the values remained significantly higher in the phobic group than in the controls. Similar results were found for STAI scores. A slightly higher salivary cortisol level was found in the phobic group. No changes occurred in cortisol or protein concentrations. The salivary secretion rate increased in the phobic patients after psychotherapy.

**Conclusions:**

It could be concluded that psychotherapy is effective in the treatment of dental phobic patients.

**Trial registration:**

This study has been retrospectively registered in the German Clinical Trials Register (# DRKS00009552) on 10/19/15.

**Electronic supplementary material:**

The online version of this article (doi:10.1186/s12903-016-0264-2) contains supplementary material, which is available to authorized users.

## Background

Dental anxiety is a widespread state among the population. A recent study showed that 67 % of patients associated visits to a dentist with anxiety [1], and 11 % suffer from dental phobia [2]. Each anxiety state is connected with physiological changes, which always result in specific reactions of the body [1]. The terms “dental anxiety” and “dental phobia” have been used synonymously [3]. Today, dental anxiety is distinguished from dental phobia [4]. According to the international classification of diseases (World Health Organization ICD-10 Version 2015), dental phobia is an acknowledged disease accompanied by disproportionate and sickening fear of objects and situations that leads to avoidance behavior, which often results in cancellation of dental appointments [5]. Three reasons are known for the development of dental phobia [6, 7]. The main reason is the pain-related invasive experience during dental treatment [2, 8, 9]. The second reason is learning by copying. In an investigation of 28 dental phobic patients, 22 patients said that at least one parent was a dental phobic patient as well [10] or they noticed their mothers suffering from pain during dental treatment. The third reason is learning by instruction. Parents warn children that dental treatment is painful. One third of dental phobic patients develop their phobia between the ages of 15 and 16 years old and the other two thirds between the ages of 18 and 26 years old [8].

Dental phobia has severe effects on the oral health status of the patients because over years those patients avoid visits to the dentist even avoid oral hygiene procedures [11, 12]. In contrast to dental anxiety, there is a need for psychotherapy in patients with dental phobia prior to dental treatment [4, 13, 14]. However, there is only scarce knowledge about the outcome of the psychotherapeutic treatment.

The diagnosis of dental phobia is essential because it influences the relationship between the patient and the dentist and has an impact on oral health [12]. Oral health, in turn, has a great impact on personal quality of live [15]. Subjective dental phobia is accompanied by objective physiological symptoms (increased muscle tension, tachycardia, sweating, stomach pain; [11]), and motor symptoms (motionlessness, flight behavior, panicking; [1]). It is possible to measure the subjective anxiety level with the dental anxiety scale (DAS) questionnaire [16–18]. The DAS allows for the distinguishing of patients into three categories: no anxiety, moderate anxiety and high anxiety. Dental phobic patients belong to the third category, and they suffer from the aforementioned symptoms. The State Trait Anxiety Inventory (STAI; [19, 20]) differentiates two anxiety states: the state anxiety, which is a temporary emotional condition, and trait anxiety, which is anxiety as a personal trait. The trait model is used to compare persons or groups of persons; the state model measures the effects of stress in various situations. To measure STAI state values, a baseline is determined before stress induction and is compared with the values after stress induction [18–21].

Another possibility for measuring the anxiety level is the determination of the salivary cortisol level [22, 23]. Recent studies have shown that the salivary cortisol level increases after acute short-term stress [23–25]. The salivary protein concentration and salivary flow rate also play important roles in diagnosing anxiety, as well as in overall oral health [24–27]. Therefore, these parameters are also important markers for quantifying dental phobia.

Psychotherapy for dental phobia comprises three different alternative approaches: confrontation therapy, stress coping and cognitive restructuring [4, 28]. Confrontation therapy has been shown to be the most successful. With this therapy, the patients are confronted repeatedly with the anxiety-producing stimulus, which leads to a reduction in anxiety [28–30]. In stress coping therapy, patients learn to recognize anxiety-stimulating situations and how to address them using mental and physical relaxation exercises [4]. The cognitive restructuring method uses videos of dental treatment situations to provoke negative feelings, which are used to develop alternative, more realistic thinking patterns [30, 31].

The main question of this study was to explore the effects of psychotherapy in patients with dental phobia on their psychological and physiological parameters.

## Methods

**Fig. 1:**
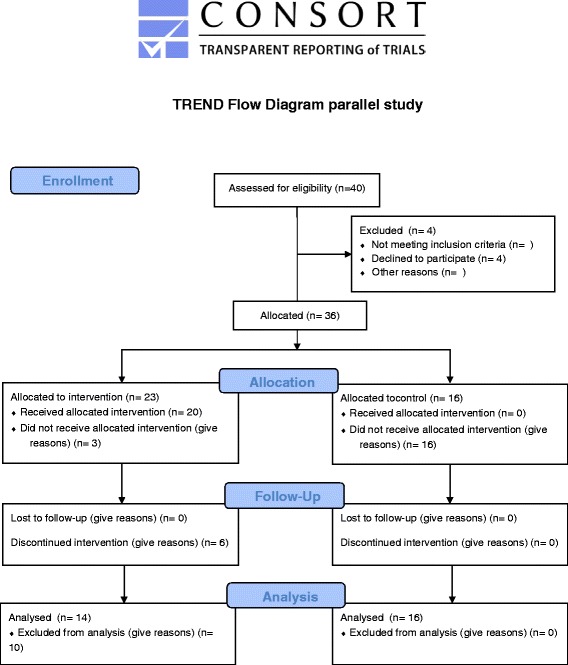
Consort Flow chart of the patient allocation

A power calculation, based on the data of a previous study [25], was performed with a power of 0.8 and α = 0.5. The power analysis revealed a minimum sample size of 13 subjects. As program for the power analysis Axum 7 (Mathsoft, Cambridge, Massachusetts, USA) was used. Twenty-four patients were included in the psychotherapy group and 16 in the control group. Of the 24 patients in the phobic group 1 did not meet the inclusion criteria. Of the remaining 23 patients three patients refused participation. Twenty patients entered the psychotherapy. Finally 14 patients finished the psychotherapeutic treatment and could be followed up until saliva collection at T3. The whole procedure is listed in Fig. 1.

### Selection of patients and determination of anxiety levels

The patients suffering from dental phobia were recruited from the Bochum dental clinic, and the control group was recruited from the dental clinic of Witten/Herdecke University. Patients suffering from dental phobia were aware of their problem and visited the dental clinic because it specialized on the treatment of those patients. All patients who visited the clinic did not suffer from acute pain, xerostomia had no chemotherapy or radiatio. At each visit, all the patients were asked to answer the State Trait Anxiety Inventory (STAI-State) [21] and the dental anxiety scale (DAS) [16–18] to determine their psychological anxiety levels. The indication for psychotherapeutic interventions was based on a DAS score > 38 in combination with avoidance of dental treatment. In addition, the Short Form Health Survey (SF-36) was used to determine the impact of dental phobia on the patients’ wellbeing. The objective anxiety level was determined using the measured salivary cortisol level.

### Study design

The first salivary sample (T0) was collected immediately after the patients entered the clinic. The patients were informed that at this visit only an initial oral investigation would be carried out. The second sample was collected after an initial oral investigation (determination of the DMF-T index; T1) during the same visit. Saliva was collected in sitting position in a quiet surrounding. In the phobic group, psychotherapeutic treatment followed. The psychotherapy lasted at least 3 weeks with weekly sessions of 1 h. After finishing the psychotherapeutic treatment, the patients visited the dental clinic again, and a third collection of salivary samples followed (T2) after the initial oral investigation. After 3 months, another salivary sample was collected (T3) at the final visit to determine the lasting effect of the psychotherapeutic intervention.

### Psychotherapy

The psychotherapy consisted of an initial anamnestic exploration to determine the subjective reasons for the phobia. The patient was to obtain a sense of his or her physiological and psychological reactions to develop strategies for coping with stress [1]. In addition, a CD was given to the patients that described active muscle and stress relaxation. In the second session, experiences were discussed, and the results of the dental cognition questionnaire [32] were evaluated. The patients had to develop positive thoughts to overcome the situations of anxiety and develop active coping strategies. In the third session, the patients watched a video demonstrating a dental treatment and discussed signs of relapse and how to cope with them.

### Saliva collection and determination of biochemical parameters

Saliva was collected in a silent atmosphere with the subject in a sitting position and spitting saliva into a plastic tube for 5 min. The total amount was weighed, and the secretion rate per minute was calculated. Then, the samples were frozen at −80^0^ until further use. Before use, the saliva samples were centrifuged for 1.5 min at 525 g. Cortisol concentrations were measured using an enzyme-linked immunosorbent assay (ELISA; RE 52611, IBL International, Hamburg, Germany). Protein concentrations were determined with Coomassie Brilliant Blue G-250 dye [33].

### Statistics

Primary endpoint of the investigation was the comparison between the measurement at T0, T2 and T3. Secondary endpoints were the DAS scores, STAI-state scores, salivary secretion rate, protein concentration and salivary cortisol concentration. As the Kruskal-Wallis test revealed no normal distribution of the data comparison was calculated with the non-parametric Wilcoxon signed-rank for related variables within the groups. Bonferroni adjustment of the α error of 0.05 was done and resulted in a new *p* value of p 0 0.025. Statistical comparison between the groups was calculated with the non-parametric Mann-Whitney *U* test for independent variables. As statistical program served statistical package for social sciences (SPSS, Rel. 21, IBM Corporation, Amronk, NY, USA).

## Results

**Fig. 2:**
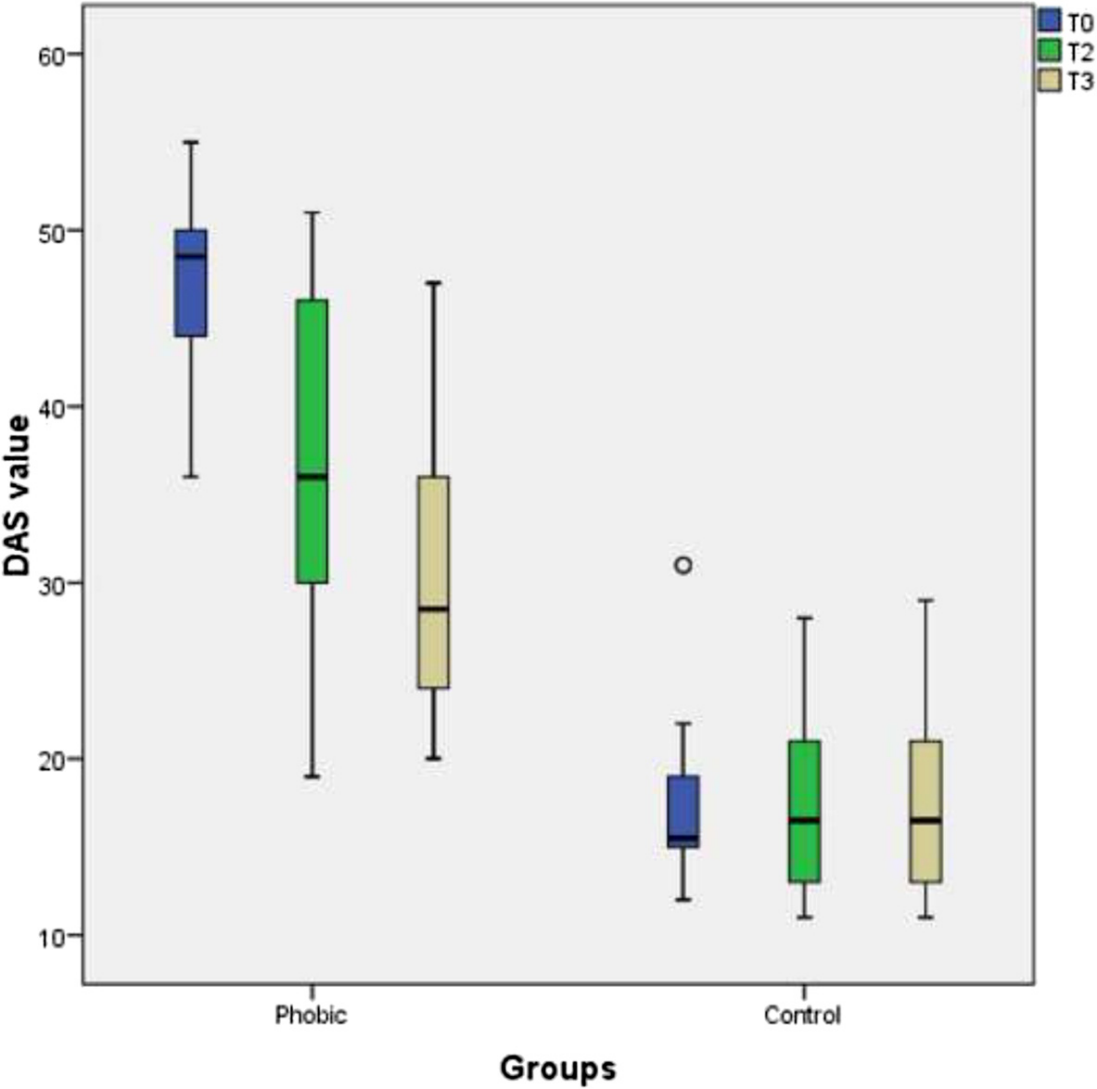
Boxplot graphics of the DAS values of the dental phobic patients before and after psychotherapeutic treatment and of the control patients. The score difference before and after treatment was statistically not significant (*p* = 0.028) at T1 but is was significant at T3 (*p* = 0.007). The difference between the phobic group and the control group was significant (*p* < 0.001)

There was no significant difference in the DAS at T1 after psychotherapeutic treatment (*p* = 0.028). At T3 the difference in the DAS scores between both groups was significant (*p* = 0.007). However, the DAS level remained significantly (*p* < 0.001) higher in the phobic group than that in the control group (Fig. 2). The data of the descriptive statistics are summarized in Table 1.

**Table 1:** Descriptive data of the DAS values

collection time point		T0	T2	T3		T0	T2	T3	
Median		48.5	36	28.5	Phobic	15.5	16.5	16.5	Control
Minimum		36	19	20		12	11		
Maximum		55	51	47		31	28		
Quartile	25 %	43	30	23.5		15	13	37	
	75 %	50.25	46.25	12.75		19.75	21.5	21	
*p* value			0.028	0.007			0.327	0.162	

**Fig. 3:**
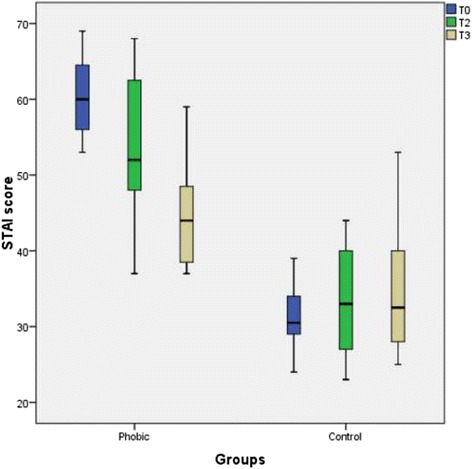
Boxplot graphics of the STAI state score values of the dental phobic patients before and after psychotherapeutic treatment and of the control patients. The score difference before and after treatment was statistically insignificant at T2 (*p* = 0.15) but it was significant at T3 (*p* = 0.018). The difference between the phobic group and the control group was statistically significant (*p* = 0.001). The STAI score in the phobic group after psychotherapeutic treatment remained significantly higher than that in the control group

Similar results as for the DAS score were found for the STAI state score values. No singinificant difference was found at T2 (*p* = 0.15) but at T3 (*p* = 0.018). The difference in the STAI state score values was significant at all time points (*p* < 0.001) (Fig. 3). The data of the descriptive statistics are summarized in Table 2.

**Table 2:** Descriptive data of the STAI score values

collection time point		T0	T2	T3		T0	T2	T3	
Median		60	33	44	Phobic	52	2	32.5	Control
Minimum		53	23	37		37	23	25	
Maximum		69	44	59		68	44	53	
Quartile	25 %	54	28.25	38.5		46	27	28	
	75 %	67	34	48.5		65	40.25	40	
*p* value			0.15	0.018			0.041	0.02	

**Fig. 4:**
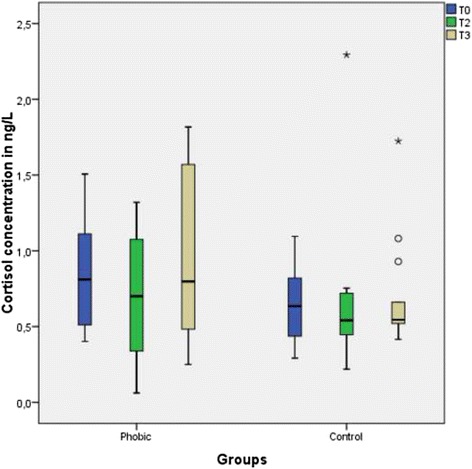
Boxplot graphics of the salivary cortisol concentrations of the dental phobic patients before and after psychotherapeutic treatment and of the control patients. The score difference before and after treatment was not statistically significant in either group. The difference between the phobic group and the control group was also not significant

Overall the salivary cortisol concentration was slightly higher in the phobic group than in the control group (Fig. 4), but the difference was not significant. After psychotherapy, the cortisol level decreased slightly but not significantly. The data of the descriptive statistics are summarized in Table 3.

**Table 3:** Descriptive data of the salivary cortisol concentration

collection time point		T0	T2	T3		T0	T2	T3	
Median		0.88	0.739	0.797	Phobic	0.634	0.54	0.545	Control
Minimum		0.401	0.062	0.25		0.291	0.218	0.415	
Maximum		2.2	1.319	1.816		1.095	2.292	1.724	
Quartile	25 %	0.518	0.411	0.477		0.427	0.441	0.515	
	75 %	1.154	0.906	1.624		0.838	0.725	0.728	
*p* value			0.14	0.575			0.331	0.683	

**Fig. 5:**
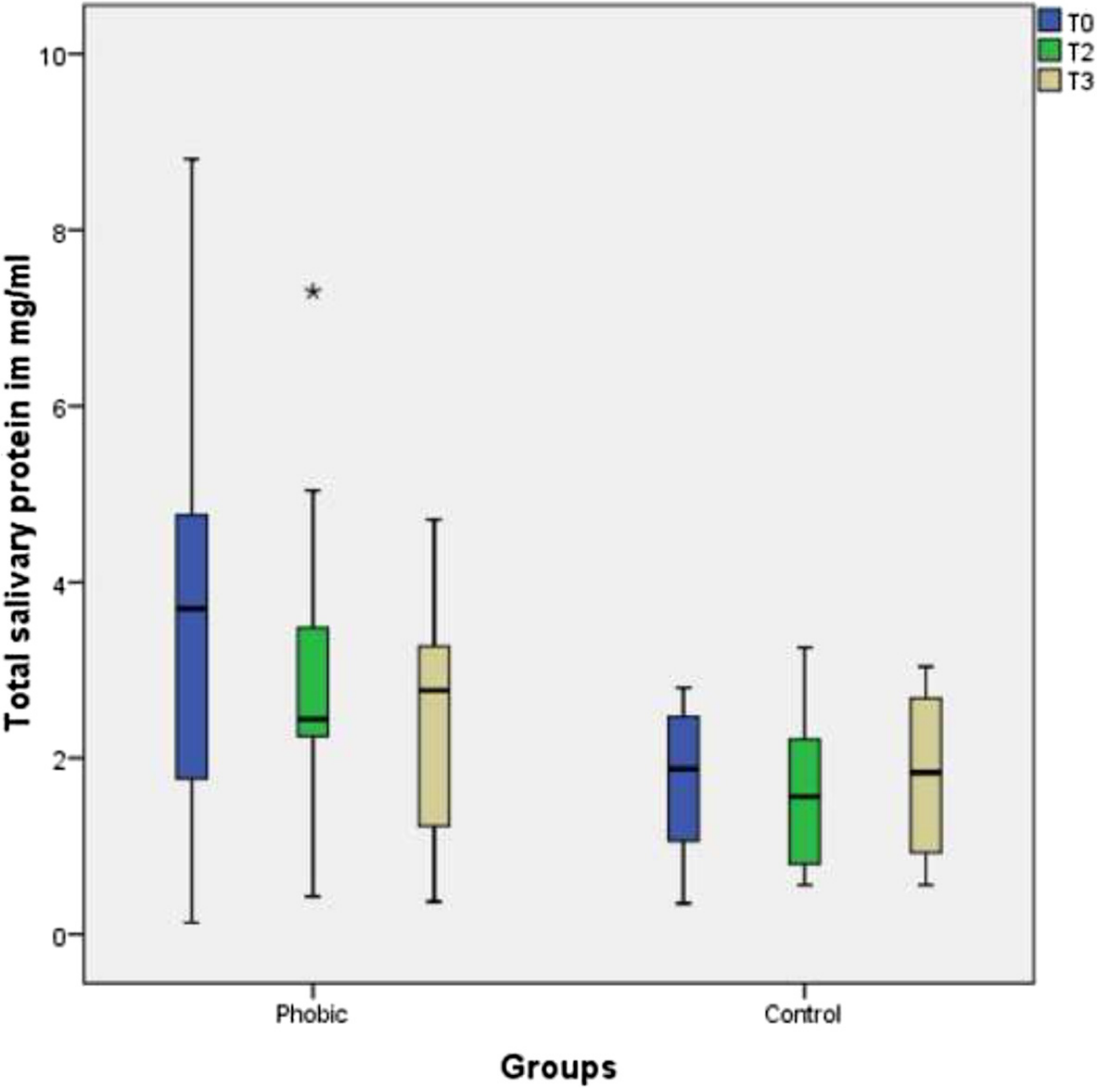
Boxplot graphics of the salivary protein concentrations of the dental phobic patients before and after psychotherapeutic treatment and of the control patients. The local difference in the protein concentration before and after treatment was not statistically significant in either group. The difference between the phobic group and the control group was also not significant

No significant changes in the salivary protein concentrations occurred after psychotherapy, and no differences were detected between the phobic group and the control group (Fig. 5). The data of the descriptive statistics are summarized in Table 4.

**Table 4:** Descriptive data of the salivary protein concentrations

collection time point		T0	T2	T3		T0	T2	T3	
Median		3.285	2.32	2.77	Phobic	1.88	1.565	1.84	Control
Minimum		0.13	0.16	0.37		0.35	0.56	0.56	
Maximum		8.81	7.3	4.71		2.8	3.26	3.04	
Quartile	25 %	1.45	1.6	0.51		1.05	0.777	0.885	
	75 %	4.64	3.37	3.37		2.48	2.41	2.685	
*p* value			0.272	0.091			0.875	0.509	

**Fig. 6:**
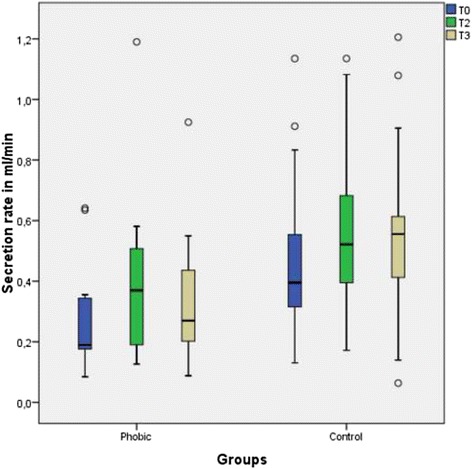
Boxplot graphics of the salivary secretion rate of the dental phobic patients before and after psychotherapeutic treatment and of the control patients. The difference in the secretion rate before and after psychotherapeutic treatment was not significant (*p* = 0.026). There was a significant difference in salivary secretion rate between the phobic group and the control group at T0 (*p* = 0.010).between the control and phobic groups. At T2 and T3 this difference was insignificant (T2 *p* = 0.21 and T3 *p* = 0.044)

The salivary secretion rate after psychotherapeutic treatment of the dental phobic patients increased markedly and was similar to that of the control group. (Table 5 and Fig. 6). At T0 a significant difference was found between the phobic group and the control group (*p* = 0.010). At T2 and T3 no significant difference could be observed (p 0.21 at T2 and *p* = 0.044 at T3).

**Table 5:** Descriptive data of the salivary secretion rate

collection time point		T0	T2	T3		T0	T2	T3	
Median		0.186	0.406	0.269	Phobic	0.395	0.521	0.555	Control
Minimum		0.084	0.126	0.087		0.13	0.171	0.063	
Maximum		0.641	1.225	0.925		1.134	1.135	1.205	
Quartile	25 %	0.133	0.166	0.172		0.31	0.384	0.386	
	75 %	0.363	0.586	0.542		0.623	0.718	0.686	
*p* value			0.01	0.328			0.21	0.14

The investigation of the DMFT index showed significantly (*p* < 0.001) increased values in the phobic group (17.36 ± 5.98) compared to the control group (2.56 ± 1.99).

## Discussion

The increased DAS score in the phobic group clearly demonstrated an increased anxiety level compared to the control group. After psychotherapeutic treatment, the DAS score in the group of dental phobic patients decreased, which showed that the psychotherapeutic intervention was successful, in accordance with the results of earlier studies [13, 14]. However, in comparing the dental phobic group with the control group, the DAS values of the phobic group remained significantly higher, indicating a limited success of the psychotherapy without equaling the control group values. This outcome led to the conclusion that only three psychotherapeutic sessions and the additional exercise of muscle relaxation would induce successive approximation of the phobic stress reaction to that of the control group. We concluded that this short-term psychotherapeutic intervention was useful for phobic patients, who could return to dental practices and undergo dental interventions without long-term latency after the initial investigation. An interesting finding was that at T3 the DAS scores and the STAI state scores were even better than at T2 immediately after the psychotherapeutic intervention.

The results of the STAI state values were similar to those of the DAS values, with a slightly decreased score value after psychotherapy. However, this difference was not significant. Isolated analysis of the STAI state score showing no differences before and after the psychotherapeutic intervention could be interpreted as a mismatch between the investigation instrument used and the stress characteristics, such as phobia in this case. Nevertheless, the STAI state score could be used as a screening instrument for dental phobia, as shown by the significantly increased values for T0 and T2 and T3 in the phobic group compared to the control group. We concluded that the STAI state could not be used for longitudinal comparative studies in cohorts with dental phobia. Taken together, both of the measurements, the DAS and the STAI state, seemed to be valuable for the determination of anxiety level.

Both psychologic indicators the DAS score and the STAI state score showed significant differences at T3 in comparison with T0. This might be interpreted as a long term effect of the psychotherapeutic intervention. A limitation of this study is the relative high dropout rate of the phobic patients. As dental phobic patients usually avoid the visit of a dental office {Armfield, 2009 #1583;Willershausen, 1999 #1703}, they may also avoid psychotherapy, because during this therapy they are confronted with the situation in the dental office.

The salivary cortisol concentration in the dental phobic group was slightly higher than that in the control group, but not significantly. These findings are in accordance with another study which showed in social phobic patients a trend towards higher cortisol levels but not significant [23]. In contrast, in cases of acute mental anxiety, the salivary cortisol concentration significantly increased [25]. An explanation for this difference might be that dental phobia is more or less chronic stress, which might have a different impact on cortisol secretion and the plasma cortisol level. However, there are other studies which found no correlation between dental anxiety and the salivary cortisol level [34, 35]. Furthermore, there are not many studies which clearly distinguished between dental anxiety and dental phobia. Controversial results have been reported about the correlation of salivary cortisol and differing stress situations {Judd, 2016 #1888;Trueba, 2016 #1889}. The impact of chronic and acute stress may have different effects on the salivary cortisol level.

The slightly increased but not significant differences in protein concentrations between the dental phobic group and the control group were not in accordance with the results of other studies, which showed a significantly increased protein concentration in acute mental stress subjects [24, 25, 36]. Here, the explanation might also be that dental phobia is not an acute anxiety state.

Another study found that in subjects with acute mental stress, the salivary secretion rate was not reduced [24]. In contrast with acute mental stress patients, dental phobia induced a decrease of the salivary secretion rate. Regarding the positive effect of the psychotherapeutic intervention measured by DAS, we concluded that diminishing the patients’ anxiety state led to equalization of their salivary secretion rate to that of the control group and to improvement of the acceptance by phobic patients of dental investigation and treatment, which might have long-term positive effects on the DMFT score. Therefore, longitudinal studies to investigate this aspect are needed.

The results of the DMFT score comparison are reinforcing earlier studies [12]. In addition to the anxiety-associated avoidance of dental treatment, the diminished salivary secretion rate in patients with dental phobia could be one possible explanation for the increased DMFT score, as the results of earlier studies have shown [37].

## Conclusions

From the results, it might be concluded that according to the DMFT score, patients with dental phobia have reduced oral health. Furthermore, psychotherapeutic treatment of dental phobia is effective in reducing the anxiety state and improving the acceptance of dental treatment by phobic patients. However, it does not reach the level of non-phobic patients. Other than in acute mental stress, the salivary parameters and the cortisol and protein concentrations do not reflect changes in the anxiety state in this study. Psychotherapeutic treatment increases the salivary secretion rate in phobic patients to that in non-phobic patients and improves oral health. Therefore, measurement of the salivary secretion rate might be a good indicator for the success of the treatment. Also, STAI state determination might be a useful tool for the recognition of patients with dental phobia, although it is not possible to measure the long-term effects of the psychotherapeutic intervention. The DAS, STAI state and salivary secretion rate are useful markers for the recognition of patients with dental anxiety. Saliva is a sensitive stress marker for dental anxiety.

## Abbreviations

DAS, dental anxiety score; DMF-T, decayed, missing, filled teeth; ICD, international classification of diseases; SF-36, Short Form Health Survey; STAI, state trait anxiety score

## Additional file

Additional file 1: Statistik Zahnarztphobie 2015-01-18.(XLSX 16 kb)
